# Avian Ultraviolet/Violet Cones Identified as Probable Magnetoreceptors

**DOI:** 10.1371/journal.pone.0020091

**Published:** 2011-05-25

**Authors:** Christine Nießner, Susanne Denzau, Julia Christina Gross, Leo Peichl, Hans-Joachim Bischof, Gerta Fleissner, Wolfgang Wiltschko, Roswitha Wiltschko

**Affiliations:** 1 Fachbereich Biowissenschaften der J.W. Goethe-Universität Frankfurt, Frankfurt am Main, Germany; 2 Max-Planck-Institut für Hirnforschung, Frankfurt am Main, Germany; 3 Fakultät Biologie, Universität Bielefeld, Bielefeld, Germany; Lund University, Sweden

## Abstract

**Background:**

The Radical-Pair-Model postulates that the reception of magnetic compass directions in birds is based on spin-chemical reactions in specialized photopigments in the eye, with cryptochromes discussed as candidate molecules. But so far, the exact subcellular characterization of these molecules in the retina remained unknown.

**Methodology/Principal Findings:**

We here describe the localization of cryptochrome 1a (Cry1a) in the retina of European robins, *Erithacus rubecula*, and domestic chickens, *Gallus gallus*, two species that have been shown to use the magnetic field for compass orientation. In both species, Cry1a is present exclusively in the ultraviolet/violet (UV/V) cones that are distributed across the entire retina. Electron microscopy shows Cry1a in ordered bands along the membrane discs of the outer segment, and cell fractionation reveals Cry1a in the membrane fraction, suggesting the possibility that Cry1a is anchored along membranes.

**Conclusions/Significance:**

We provide first structural evidence that Cry1a occurs within a sensory structure arranged in a way that fulfils essential requirements of the Radical-Pair-Model. Our findings, identifying the UV/V-cones as probable magnetoreceptors, support the assumption that Cry1a is indeed the receptor molecule mediating information on magnetic directions, and thus provide the Radical-Pair-Model with a profound histological background.

## Introduction

The magnetic compass of birds was first described in the 1960s for European robins, *Erithacus rubecula*, a small passerine migrant [1]. Since then, this compass mechanism has been demonstrated in a variety of migratory and non-migratory birds of different orders [2], among them the domestic chickens, *Gallus gallus* [3]. According to behavioral experiments, it is an ‘inclination compass’ based on the axial course of the field lines rather than on their polarity, and it is light-dependent, requiring light in the short-wavelength part of the spectrum (see [2] for review).

How birds perceive magnetic directions remained largely unknown. Several hypotheses were forwarded; the one presently favored is the Radical Pair Model by Ritz and colleagues [4]: it proposes that photon absorption in specialized receptor molecules leads to an electron transfer and the formation of radical pairs. These occur in two states, singlet and triplet, which are in a chemical balance that depends on the alignment of the receptor molecules in the magnetic field. To obtain information on magnetic directions, the singlet or triplet yield in the various spatial directions would have to be compared. Hence, for a magnetic compass based on radical pair processes, three crucial requirements must be fulfilled: (1) light has to reach the receptor molecules to induce the formation of radical pairs, (2) the arrangement of the receptor cells has to cover all spatial directions to allow the comparison of the respective singlet or triplet yields, and (3) within any one receptor cell, all receptor molecules have to be aligned in the same direction to act as a functional unit. The eye with its spherical shape meets the first conditions and was therefore suggested as site for magnetoreception [4]. This has been supported by experimental evidence: magnetoreception is indeed mediated by the eye [5], and, by using radio frequency fields as diagnostic tools, the underlying mechanism could be identified as radical pair process [6]–[8].

Ritz and colleagues [4] already discussed cryptochromes as suitable candidates for the receptor molecules. Cryptochromes are blue light-sensitive flavoproteins that can form radical pairs [9], [10]; they are related to the photolyases which catalyze DNA repair in plants via electron transfer [11]. Cryptochromes were first identified in plants, but then also found in animals, where they are e.g. involved in the circadian clock [12], [13]. Cryptochrome-controlled processes were found to be affected by magnetic fields [14], [15], indicating that this molecule has the potential to mediate magnetic information. A study subjecting birds to different radio-frequencies and intensities revealed features of the receptor molecule that would be met by cryptochrome [8].

Cryptochromes have been reported in the retina of several bird species, among them domestic chickens [16] and migratory passerines [11], [17], [18]. Two forms of cryptochrome 1, Cry1a and Cry1b, which are splice products of the same gene, and cryptochrome 2 were identified [17]. Yet presence in the retina is only one prerequisite for cryptochrome to serve as receptor molecule for magnetic compass information; the other requirements mentioned above must also be met. It is therefore important to determine which of the cryptochromes forms the crucial radical pairs and where within the eye and also where inside the respective cells this cryptochrome is located.

Cryptochrome 2 seemed a less plausible candidate, because its sequence contains a nuclear localization signal [17], [18], which is not characteristic for a receptor molecule. The two forms of cryptochrome 1, in contrast, are cytosolic. They are not trans-membrane proteins, but they could be fixed to a membrane or to the cytoskeleton to keep them in a specific alignment. We therefore focused our search on the two forms of cryptochrome 1. In the present paper, we report that Cry1a is located in the retina in a way that is in accordance with a function as magnetoreceptor molecule [4]. Our study involves two bird species, domestic chickens and European robins. The two are not closely related, show marked differences in behavior and habitat, but have the same type of magnetic compass mechanism [2], [3].

## Results

Quantitative comparison of the immunolabeling intensity of retinae sampled at different times of day and at different seasons did not indicate any differences between these samples. Furthermore, there were no obvious difference between the right and the left eye. This was true for chickens and robins alike. Hence we here show only one representative example of each of the data sets.

**Figure 1:**
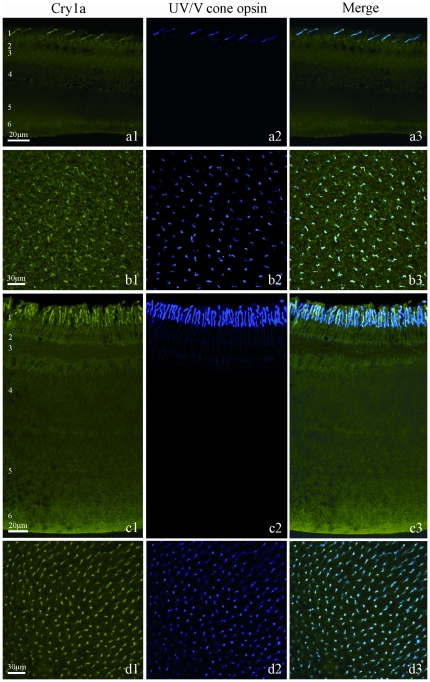
Immuno-labeling for Cry1a and UV cone opsin, and their co-localization in the retina. (**A**), Vertical section of chicken retina; (**B**), whole mount of chicken retina; (**C**), vertical section of European robin retina; (**D**), whole mount of robin retina. The different layers in the vertical sections are indicated: 1 outer and inner segments of the photoreceptors with the oil droplets in between; 2 outer nuclear layer; 3 outer plexiform layer; 4 inner nuclear layer; 5 inner plexiform layer; 6 ganglion cell layer. Left column: (**A1** to **D1**): Cry1a immunofluorescence (rendered in green) is inside the outer segment of a very slender photoreceptor type. Middle column: (**A2** to **D2**): UV/V cone opsin immunofluorescence (rendered in blue) in the same section. Right column: (**A3** to **D3**): Merge of the images, indicating that Cry1a and the UV/V cone opsin co-localize. In robins, the the population density of the Cry1a/UV appers to be higher than in chickens.

The results in both species were the same: Cry1a was found in one specific population of very slender photoreceptors of the single cone type (Fig. 1). Double labeling with the Cry1a antiserum and the UV/V opsin antiserum showed that this receptor is the UV/V (SWS1) cone, which has a higher population density in robins than in chickens (Fig. 1). Birds have four color cone types [19]; the long wavelength-sensitive (LWS) cones did not contain Cry1a labels and the same appears to hold for the other two cone types (SWS, MWS). Cry1a was found only in UV/V cones, and all these cones contained Cry1a in chickens as well as in robins. Avian cone outer and inner segments are clearly separated by an oil droplet; in these cones, Cry1a label was present in the outer, but not in the inner segment (Fig. 1A,C). Since Cry1a occupied a smaller region within the outer segment than the opsin, it may appear as if in the section shown in Fig.1C some UV/V cones do not contain Cry1a. However, focusing through the section and the flat view in the whole mounts clearly showed that Cry1a label was present in every UV/V cone. The topographic distribution of the Cry1a-containing cone was assessed in flattened whole retinae, showing that these cones were present across the entire retina in both species, with no obvious density peaks (Fig. 1B,D).

**Figure 2:**
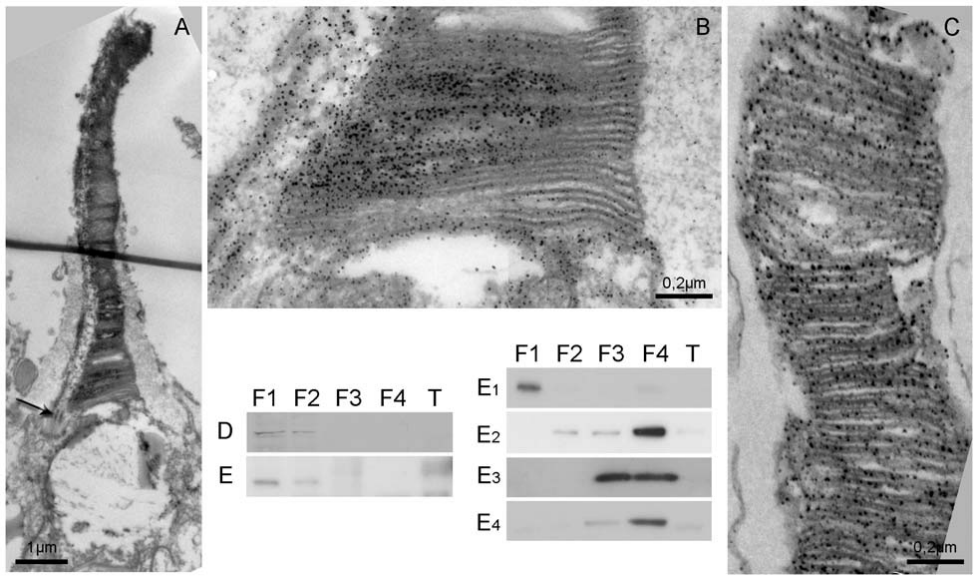
Electron-microscopic image and Western blots. (**A**) Outer segment of a long, slender cone photoreceptor of the chicken retina, with the large oil droplet visible at the base and the connecting cilium (marked by the arrow) on the left. Cry1a, labeled with diaminobenzidin and silver intensification, is visible as dark dots along the disc membranes. (**B**) Higher magnification of the lower part of the outer segment in (A). Cry1a is found along some, but not all disks. At the left side with the connecting cilium, Cry1a is transported to the outer segment, where it is to bind to the membranes. (**C**) Outer segment of a cone photoreceptor of the robin retina, also showing ‘bands’ of Cry1a label. Western blot of robin (**D**) and of chicken retina (**E**), respectively. F1, cytosolic fraction; F2, membrane fraction; F3; nuclear fraction, F4, cytoskeletal fraction, T, tongue tissue from the same bird as control. Cry1a is found in the cytosolic and the membrane fraction in both species. Markers for the different fractions shown for chicken: (**E1**) Protein Kinase C for cytosolic, (**E2**) E-cadherin for membrane, (**E3**) Histon H3 for nuclear in chicken, (**E4**) Actin for cytoskeletal fractions (35). The markers show that F1 and F2 are free from other fractions (see Protein Kinase C and E-cadherin). E-cadherin is also bound to the actin cytoskeleton in F4, but a low ‘spill-over’ in F3 is visible. The same is true for Actin, the control for the cytoskeletal fractions. Histon H3 is in F3 but also in F4, because of its high abundance in the cell lysate.

A further demand to act as receptor molecule for magnetic directions is a uniform alignment of the Cry1a molecules within the receptor cell. The outer segment is composed of disc membranes, a highly ordered structure offering a scaffold for such an orderly alignment. Immuno-electronmicroscopy was used to assess whether Cry1a is associated with structures of the outer segment. In electron micrographs, the oil droplet between the inner and outer segment of the cones was visible (Fig. 2A). Cry1a label was found in highly ordered bands alongside some disc membranes of the outer segment of a special very slender cone type (Fig. 2A,B,C), that morphologically corresponds to the UV/V cone identified by light microscopy. At the base and in the middle of the outer segment, Cry1a-labelled zones alternated with unlabelled zones, which resulted in a striped pattern. The observed orderly array suggests that Cry1a could be bound to the membranes. Another aspect of the localization of Cry1a in the outer segment is also visible in the electron micrographs: Cry1a label was extending to the outer membrane limiting the receptor on the side of the connecting cilium where the proteins are transported from the inner to the outer segment, but not on the opposite side (Fig. 2B). Like in the light microscopic staining, Cry1a was immuno-labeled only in the outer segment, not in the inner segment (see Discussion and supplemental Fig. S1).

The ordered arrays of Cry1a along the membrane discs in the outer segment seen at the electron microscope indicated that this protein could be membrane-bound. Hence we subjected chicken and robin retinae to a differential cell fractionation protocol. The increasing dissolving strength of the buffers separates cytosolic, membrane, nuclear and cytoskeleton fractions. In both species, Cry1a was detected in the cytosolic and membranous fraction (Fig.2 D,E). In line with the electron microscopy data, this suggests that soluble, cytosolic Cry1a is recruited and then probably bound to membranes.

## Discussion

The present study demonstrates that Cry1a is located in the outer segment of all UV/V cones in the retina of chickens and robins. This cone type is distributed across the entire retina. The ultra-structural analysis suggests that Cry1a is associated with the disc membranes, which is supported by the subcellular fractionation data showing Cry1a in the membrane fraction in both species.

### Location of Cry1a

Light and electron microscopic immuno-labeling showed Cry1a in the outer segments of the UV cones, but not inside the inner segment, where all proteins, including Cry1a and the opsins, are synthesized. Possibly, Cry1a is present in the inner segment only in concentrations too low for detection by the anti-Cry1a antiserum, or it is in a configuration that does not allow the antiserum to bind. Similarly, the UV/V opsin was immuno-labeled in the outer segment only, so this may not be an uncommon phenomenon. Alternatively, Cry1a formation in the inner segment and its transport to the outer segment did not take place at the time of retina fixation. Rod and cone outer segment renewal, by disc addition at the basal end and disc shedding at the apical end, is known to underlie a circadian rhythm. This could also explain the Cry1a bands seen in the outer segment, reflecting alternating active and inactive phases of moving Cry1a to the disks. Young [20] estimated chicken cones to renew ca. 40 discs per day, with a peak renewal phase early in the dark period (see also [16]). The Cry1a bands in our material have a closer spacing than 40 discs, which may suggests several daily production peaks, or a different renewal rate in UV/V cones.

We found no indications that Cry1a is released from the outer segment. Apparently, Cry1a is not transported from the photoreceptor to the pigment epithelium, as is the retinal of the visual pigments – retinal cannot be re-isomerized within the photoreceptor cells, but has to be transported into the pigment epithelium for regeneration. In case of magnetoreception by cryptochrome, the situation is different insofar as not photoreduction, but re-oxidation appears to be the crucial reaction mediating compass information [8]. Hence the re-oxidation process has to take place within the photoreceptor to induce an output signal. At the apex, however, where the photoreceptor outer segment is decomposed, this involves the entire membrane with all proteins.

The Radical Pair Model of magnetoreception [4] requires that the receptor molecules within any one cell be all aligned in the same direction to act as a functional unit. This requirement appears to be met, as Cry1a seems to be arranged along the membranes of the disks. Such proteins can usually move or rotate to some degree; calculations by Hill and Ritz [21] indicate that a certain amount of movements is permitted without disrupting the receptor function.

While we find Cry1a only in the outer segments of the UV/V cones, Mouritsen et al. [18] reported cryptochrome 1 in the ganglion cell layer of migrating garden warblers, *Sylvia borin*. That study used a commercial antibody for cryptochrome 1 that would label Cry1a and Cry1b alike. Hence, rather than indicating a species difference, it seems most likely that the cryptochrome 1 signal in the ganglion cells represented Cry1b.

### The UV/V Cones as Magnetoreceptors

The observation that Cry1a is found in only one type of photoreceptor cell, the UV/V cone, was rather unexpected. Of the four avian single cone types, the UV/V cone covers the short-wavelength end of the visual spectrum. Incidentally, the so-called ‘UV/V cone’ with the SWS1 opsin is probably tuned to ultraviolet light in robins, but to violet light in chickens [19]. The UV/V cones, comprising about 9% of the cones in chickens and a somewhat different percentage in other species [22], are rather evenly distributed across the entire retina [23]. The present data confirm that the UV/V cones are regularly arranged. There is no location in the retina where UV/Cry1a-containing receptors are particularly densely packed – a special center for magnetoreception, something like a ‘magnetic fovea’, does not exist. This is in accordance with the Radical Pair Model [4] where a rather even distribution of magnetoreceptor cells over the entire retina is postulated. Assuming that the location and binding of the Cry1a protein is identical in all UV/V cones, their distribution within the spherical shape of the eye ensures that the radical pairs formed by Cry1a are in different alignments with the geomagnetic field – according to their orientation with respect to the field lines, their singlet/triplet ratio would differ. The assumed centrally symmetric activation pattern on the retina illustrated by Ritz and colleagues [4] thus does not seem unrealistic. If the birds turn their heads, this magnetic field-induced activation pattern would shift as a whole across the retina, so that the direction of the magnetic field can be detected independent of the birds' position.

The association of magnetoreception and ultraviolet/violet vision suggests an interrelationship between the two and raises the question whether magnetoreception is affected by the visually induced activity of the UV/V cones. Behavioral tests with migratory European robins under dim monochromatic light have shown that the birds were well oriented in their migratory direction under light from the entire short-wavelength range of the spectrum, indicating that their magnetic compass worked properly from below 372 nm UV to 565 nm green [24]. The spectral emission of the green diodes (LEDs) used in these studies (measured with Spectrometer 16-001293, Dual Faseroptik), does not contain any light in the range to which the UV/V cone could have responded. This indicates that magnetic directional information is mediated regardless of whether the UV cone opsin is light-activated or not – in the initial stage, magnetoreception seems to occur independent of UV vision.

Another observation supports this idea: When the intensity of monochromatic lights was increased above certain levels in the behavioral tests, the robins failed to orient properly [25], indicating an interference with their magnetic compass. This was observed under bright UV, blue and green light alike. That is, a strong activation of the UV cones, but also a strong activation of the green cones with the UV cones not activated, disrupts the function of the magnetic compass in a similar way. These effects, restricted to monochromatic lights as rather unnatural stimuli, suggest that the primary magnetoreception processes themselves are largely independent of the visual activation of the cones, and indicate interferences at higher processing levels [25].

This leads to the question of how the radical pair mechanism of Cry1a generates the signal that mediates magnetic information. Vertebrate photoreceptors have ion channels for Na^+^ and Ca^2+^ that are kept open by cyclic GMP, and the resulting dark current leads to slight depolarization. When the opsin is activated by photon absorption, the G-protein Transducin starts the signaling cascade that closes the ion channels and hyperpolarizes the receptor. To transmit information on magnetic directions indicated by the amount of Cry1a singlets or triplets, the radical pair mechanism could either independently use the signaling cascade of UV/V opsin, or it could have a separate signaling pathway that affects the state of the ion channels in the outer membrane.

### Separating Visual and Magnetic Input

Identifying the UV/V cones as magnetoreceptors raises the crucial question about the perceptual separation of visual and magnetic information. At the photoreceptor level, the activation of the Cry1a molecule is combined with that of the UV/V opsin to a single output of the UV/V cone. Consequently, mechanisms are needed to separate the two components of the common signal for further processing. Zapka and colleagues [26] recently speculated that if the detection of magnetic directions and daytime vision occurred in the same type of photoreceptors, high light-induced activation might override or mask the magnetic compass, and considered the possibility of a second receptor mechanism for magnetoreception during the day. However, when birds use their magnetic compass under ‘white’ light of high intensity with all four cone types activated, the primary processes of magnetoreception are the same radical-pair processes as at night, as indicated by the disrupting effect of radio-frequency fields [3], [6], [27].

Several mechanisms are conceivable for separating magnetically induced and visual output, which could be performed directly at the retinal level or more centrally. A comparison of the output of adjacent UV/V cones with and without cryptochrome can be ruled out, because the present study shows that every UV/V cone contains Cry1a. Yet other comparisons, e.g. with the blue cones, seem possible, as there is some overlap in the excitation range of these two cones. Too strong asymmetry of activation by the visual stimulus, e.g. if one of the receptors were strongly activated and the other hardly at all, would hamper this comparison. Yet the wiring of the retinal network is highly complex, so other potential mechanisms to extract magnetic information already at the retinal level seem possible. In that case, however, the extracted magnetic information should then be transferred to the higher centers by an extra set of ganglion cells.

Alternatively, extraction of magnetic directional information could take place at higher processing levels. There is no doubt that the UV/V cones in birds are integrated fully in a tetrachromatic color system [28]. According to the Radical-Pair-Model [4], the output of the magnetoreceptors would lead to an orientation-dependent distinct pattern of differential activation which overlays the representation of the visual scene. Within the brain, candidates for processing of complete retinal images as needed for extraction of the magnetic information would be areas containing a topographic retinal projection. Two such retinotopic maps have been described [29], one within the optic tectum, where early electrophysiological recordings indicated an involvement in magnetic perception [30], and another one within the visual Wulst, where a subdivision was found to be active during magnetic orientation [26], [31]. In principle, extraction of magnetic information from neuronal maps could be performed on static images employing low pass filtering, because the pattern induced by the magnetic field has low spatial frequency transitions while the visual scene mostly consists of high spatial frequencies. This idea is in accordance with a recent finding that blurring of the visual image eliminating higher spatial frequencies and adding lower ones leads to a break-down of magnetic orientation [32].

Information provided during movement could also contribute, making use of image slip on the retina, called ‘optic flow’ [33]. When movement is translational, e.g. plain forward, the magnetic field-induced pattern remains the same, while the objects in the visual field are expanding and moving towards the subject. Mechanisms of separating moving and stationary objects are known from figure-ground discrimination, and neurons solving this task have been described in the optic tectum of birds [34].

### Conclusions

Our findings show that Cry1a seems to fulfill essential requirements discussed by Ritz and colleagues [4] for the receptor molecule of the magnetic compass mechanism in birds: it can form long-lived radical pairs [10], [11]; in the eye, it is exposed to light; the Cry1a proteins are associated with the disc membranes, suggesting that within one receptor cell, they are ordered in a way that they can act as a unit. The observed presence of the UV/V cones across the entire retina results in different alignments of the Cry1a molecules with respect to the magnetic field lines. With this distribution, Cry1a appears well suited to act as primary receptor molecule for the detection of directional information from the Earth's magnetic field. Also, the observation that Cry1a is always present, regardless of time of day and season, supports this role: the magnetic compass is always ready to tell the birds directions for whatever activity they may need this information.

One additional remarkable aspect is that Cry1a is restricted to the UV/V cones in both chickens and robins. This is in agreement with behavioral data indicating the same type of magnetic compass for both species [2], [3]. The two species belong to avian lineages that separated already about 95 million years ago in the late Cretaceous [35]. Hence our histological data also support the idea of a very early evolution of the avian magnetic compass, which seems to have taken place already in the Mesozoic in the common ancestor of modern birds.

## Materials and Methods

### Birds and Tissue preparation

Animal acquisition and all procedures complied with the German law and regulations on animal protection. Ethic committee approval or a special permit are not required for collecting tissues from chickens (see § 4, section 3 of the Tierschutzgesetz, the German law regulating the protection and welfare of animals). Robins were taken under permit 7922-1.62-EA 07-0005 issued by the Untere Naturschutzbehörde of the City of Frankfurt am Main (ethics and natural protection committee of the City of Frankfurt am Main).

Retinae of ten chickens and five robins were used for this study. The samples of the chickens were collected in the morning or around noon. Three samples of robins were collected at ca 18∶30 in the evening while the birds showed migratory restlessness, one at 8∶00 during migration season, and the other at 12∶00 noon about 4 weeks after the end of migration.

For both light and electron microscopy, retinae were fixed in the eyecup with 4% paraformaldehyde (PFA) in 0.1 M phosphate buffered saline (PBS, pH 7.4) for 4 h at RT and then the PFA was washed out with PBS. For light microscopic immuno-histochemistry, retinae were cryoprotected in an ascending series of sucrose solutions (10%, 20%, 30% in PBS), and embedded in tissue freezing medium (Sakura). 12–15 µm transverse sections were cut on a cryostat and mounted on Super Frost Plus slides. The slides were stored at −20°C until further processing. For whole mount light microscopic immuno-histochemistry, the material was processed soon after fixation. For electron microscopy, after fixation and PBS wash retinae were embedded in agarose, and 60–100 µm sections were cut with a vibratome. The sections were treated free floating. They were first incubated in sucrose (10%, 20%, 30%), frozen three times in liquid nitrogen and then stored at −20°C until use. For Western blot analysis, retinae were directly disintegrated in RIPA buffer or, for cell fractionation, transferred to the F1 buffer according to the ProteoExtract® Subcellular Proteome Extraction Kit manual (Calbiochem) (see below).

### Primary antibodies used for immuno-histochemistry

The following antibodies were used:

1. Guinea pig Cry1a antiserum (designed in our laboratory and produced by GENOVAC GmbH, Freiburg, Germany), raised against amino acids 601–621 of Cryptochrome 1a: (C-) RPNPE EETQS VGPKV QRQST (-N). This peptide sequence is identical in robins and chicken [17], [36]. Western blotting showed the antiserum to be specific for Cry1a in robins and chicken, other bird species were not tested.
2. Goat antiserum sc-14363 raised against a 20-aa N-terminal epitope of the human S (blue) cone opsin (Santa Cruz Biotechnology Inc., Santa Cruz, CA, USA), characterized by [37]. Phylogenetically, the mammalian blue-sensitive S cone opsin is homologous to the UV/V (SWS1) cone opsin of birds [38]; its sequence is very similar to that of the avian UV/V opsin, but not to other avian opsin sequences (see Table 1). This is supported by the fact that another antiserum against mammalian S opsin, CERN 933, also specifically labels chicken violet (SWS1) cones [39].
3. Rabbit antiserum JH492 raised against a C-terminal epitope of human M/L cone opsin (kindly provided by J. Nathans, Johns Hopkins University School of Medicine, Baltimore; see [40]. In birds, this antibody labels the LWS (red) cone opsin, but reactions with the MWS (green) cone opsin cannot be excluded.

**Table 1:** Comparison of amino acid sequences.

Opsin	Part of sequence	GenBank
Epitope recognized by antiserum sc-14363	EFYLFKNISSVGPWDGPQYH	
*Gallus gallus*, UV/V cone opsin (AA 6–25)	D**FYLF** *T* **N**G**S-V**P**GPWDGPQYH**	NP_990769.1
*Gallus gallus*, blue cone opsin (AA 13–37)	D**FY**IPMALDAPNITALSPFLV**PQ**TH	NP_990848.1
*Gallus gallus, g*reen cone opsin (AA 8–30)	N**FY**VPMSNKTGVVRSPFEY**PQ**YY	NP_990821.1
*Gallus gallus*, red cone opsin (AA 24–43)	VFTYTNSNNTR**GP**FE**GP**N**YH**	NP_990771.1
*Serinus canaria*, UV/V cone opsin (AA 5–24)	**EFYLFKN** *Q* **SSVGPWDGPQYH**	CAB91993.1
*Serinus canaria*, blue cone opsin (AA 1–19)	NLDTPNVTALSPFLV**PQ**TH	CAB91994.1
*Serinus canaria*, green cone opsin (AA 1–19)	PMSNKTGVVRSPFEY**PQ**YY	CAB91995.1
*Serinus canaria,* red cone opsin (AA 1–19)	FTYTNSNNTR**GP**FE**GP**N**YH**	CAB91996.1

The amino acid sequence of the epitope of the mammalian SWS1 opsin recognized by antiserum sc-14363 and of the most similar sequences of the cone opsins in chickens, *Gallus gallus*, and the Canary *Serinus canaria,* a passerine species are compared (cone opsins of European robins have not yet been sequenced). Sequence part of the UV/V cone opsin corresponding to the epitome recognized by the antiserum, with identical amino acids given in bold.

### Light microscopic immuno-histochemistry

For light microscopic immuno-histochemistry, retinal pigment epithelium adhering to the isolated retina was bleached using 5 ml of 1.8% NaCl in aqua dest., 4 ml of 30% H_2_O_2_, 1 ml aqua dest., 1 drop NH3 for 20–30 min [41]. This made the flat-mounted retinae transparent for microscopy without interfering with the subsequent immuno-labeling. After bleaching and washing in PBS, the retinae were pre-incubated with 10% normal donkey serum (NDS) in 0.25% Triton X-100, 2% BSA in PBS for 60 min at RT. Then the slides and the whole mounts were incubated with the primary antibodies (anti-Cry1a 1∶100, JH492 1∶10,000; sc-14363 1∶500) in 3% NDS, 0.25% Triton X-100, 2% BSA, in PBS overnight at 4°C. After washing in PBS, the tissue was incubated with appropriate secondary antibodies coupled to the fluorescent dyes CY5 and CY3 (Dianova, Hamburg) in 3% NDS, 0.25% Triton X-100, 2% BSA, in PBS for 1 h at RT. For whole mount immuno-labeling, the pecten was removed for easier preparation and the retinae were treated free floating. After staining, the retinae were mounted photoreceptor side up on Super Frost Plus slides and coverslipped with Aqua–Poly Mount (Polysciences Europe). All slides were evaluated with a confocal laser-scanning microscope (Zeiss Typ 510 META).

Several controls were performed to show the specificity of immuno-labeling. For both antibodies against the cone opsins we did only controls where we omitted the primary antibody. For anti-Cry1a we did the following controls. A first control with pre-immune serum taken before immunizing the animals showed that there were no unspecific tissue reactions by other antibodies that were already present in the immunized animals. The second control was to omit the primary antibodies from the above protocol, showing that the secondary antibodies reacted selectively with the primary antibodies and produced no artifacts. The third control was to combine guinea pig anti-Cry1a as primary antibody with an anti-goat secondary antibody, and the goat antiserum sc-14363 with an anti-guinea pig secondary antibody. This showed for the double-labeling study that there was no cross-reactivity of the primary antibodies with the inappropriate secondary antibodies. A fourth control was performed with the Cry1a antibody and the specific peptide that was used to produce the antibody. Before applying the primary antiserum on the retina, it was blocked by mixing it with this peptide. Here, any remaining label would indicate that the Cry1a antibody additionally recognizes other epitopes than the immunizing peptides, or that there are other antibodies in the serum that also bind to retinal structures. This was not the case.

The controls are shown in Figure S2 in Supporting Information.

### Pre-embedding immuno-electronmicroscopy

After pre-incubation in 10% normal goat serum (NGS) and 2% bovine serum albumin (BSA) in PBS for 60 min at RT, retinal vibratome sections were incubated with the primary antibody anti-Cry1a 1∶100 in 3% NGS, 2% BSA, in PBS over 3–4 days at 4°C. The secondary antibody was a biotinylated anti-guinea pig IgG (Vector laboratories, catalog nr. BA 7000) applied for 2 hours. Then a peroxidase-based enzymatic detection system (Vectastain Elite ABC kit; Vector) was used. For visualizing the antibody bindings, the sections were treated with 0.025% diaminobenzidine for 15 minutes. For amplification of the immune signal, a silver intensification was used [42]. The sections were incubated in 0.5% osmium tetroxide for 30 minutes at 4°C, dehydrated by an ethanol series and propylene oxide and embedded in Agar Low Viscosity Resin (Plano GmbH, Agar Scientific Limited, Essex). Ultra-thin sections (50–60 nm) were cut with Ultra S microtome (Reichert, Leica) and placed on copper grids, stained with uranyl acetate and lead citrate and evaluated with a transmission electron microscope (CM12, Philips, Hamburg). Here we also performed controls with pre-immune serum and controls without the primary antibody.

### Western blot and cell fractionation

Chicken retinae were dissociated in RIPA buffer (0.5% sodium desoxycholate, 1% Nonidet P 40, 0.1% SDS, 1 mM EDTA in PBS, supplemented with complete Protease Inhibitor (Roche) for 30 minutes on ice. Cell fractionation of robin and chicken retinae was performed with the ProteoExtract® Subcellular Proteome Extraction Kit (Calbiochem) according to the manufacturer's manual. Lysates were cleared by centrifugation, and 20 µg of protein sample were subjected to 10% SDS-polyacrylamide gel electrophoresis and electroblotted onto nitrocellulose membrane for 2 hours at 180 mA. After blocking with 5% BSA, the membranes were incubated with the guinea pig Cry1a antiserum (1∶500), followed by horseradish peroxidase-conjugated goat anti-guinea pig IgG polyclonal antiserum (Dianova, Hamburg, Germany). Immunoblots were visualized using a solution of 2.5 mM luminol, 0.4 mM p-coumaric acid, 100 mM Tris-HCl, pH 8.5 and freshly added 0.009% H_2_O_2_.

Tongue of the respective birds, a tissue without cryptochrome, was used as a control. Purity of fractions was tested by staining for proteins such as Protein Kinase C (Santa Cruz Biotechnology Inc., Santa Cruz, CA, USA) for cytosolic, E-cadherin (clone 36, BD Transduction Laboratories, Los Angeles, CA, USA) for membrane, Histon H3 (Sigma, St. Louis, MO, USA) for nuclear and Actin (Sigma Aldrich, Munich, Germany) for cytoskeleton fractions.

## Supporting Information

Figure S1 Electron-microscopic image of the inner segment of a Cry1a-immunoreactive cone in the chicken retina.
(PDF)

Figure S2 Controls for the immuno-labelling
(PDF)
