# Supplementary material for: Avian Ultraviolet/Violet Cones Identified as Probable Magnetoreceptors
Source: PLoS One. 2011 May 25;6(5):e20091. doi: 10.1371/journal.pone.0020091 (PMC3102070; doi:10.1371/journal.pone.0020091)
Supplement: Figure S1 — Electron-microscopic image of the inner segment of a Cry1a-immunoreactive cone in the chicken retina. (PDF) [file pone.0020091.s001.pdf]

Supporting Online Material

Avian Ultraviolet/Violet Cones Identified as Probable Magnetoreceptors

Christine Nießner, Susanne Denzau, Julia Christina Gross, Leo Peichl, Hans-Joachim Bischof, Gerta Fleissner, Wolfgang Wiltschko, Roswitha Wiltschko

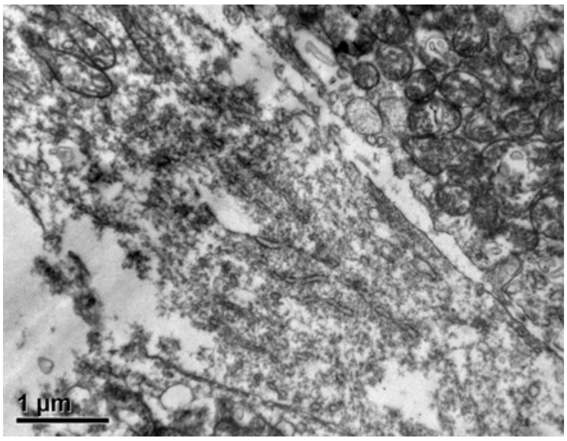

Fig. S1. Electron-microscopic image of the inner segment of a Cry1a-immunoreactive cone in the chicken retina. Extending from top left to bottom right is the Cry1a-negative inner segment of a cone having a Cry1a-positive outer segment. The inner segment of an unidentified neighbouring photoreceptor appears in the top right corner. See main text for details.
