# Supplementary material for: Avian Ultraviolet/Violet Cones Identified as Probable Magnetoreceptors
Source: PLoS One. 2011 May 25;6(5):e20091. doi: 10.1371/journal.pone.0020091 (PMC3102070; doi:10.1371/journal.pone.0020091)
Supplement: Figure S2 — Controls for the immuno-labelling (PDF) [file pone.0020091.s002.pdf]

Supporting Online Material

Avian Ultraviolet/Violet Cones Identified as Probable Magnetoreceptors

Christine Nießner, Susanne Denzau, Julia Christina Gross, Leo Peichl, Hans-Joachim Bischof, Gerta Fleissner, Wolfgang Wiltschko, Roswitha Wiltschko

Fig. 2. Controls for the immuno-labelling

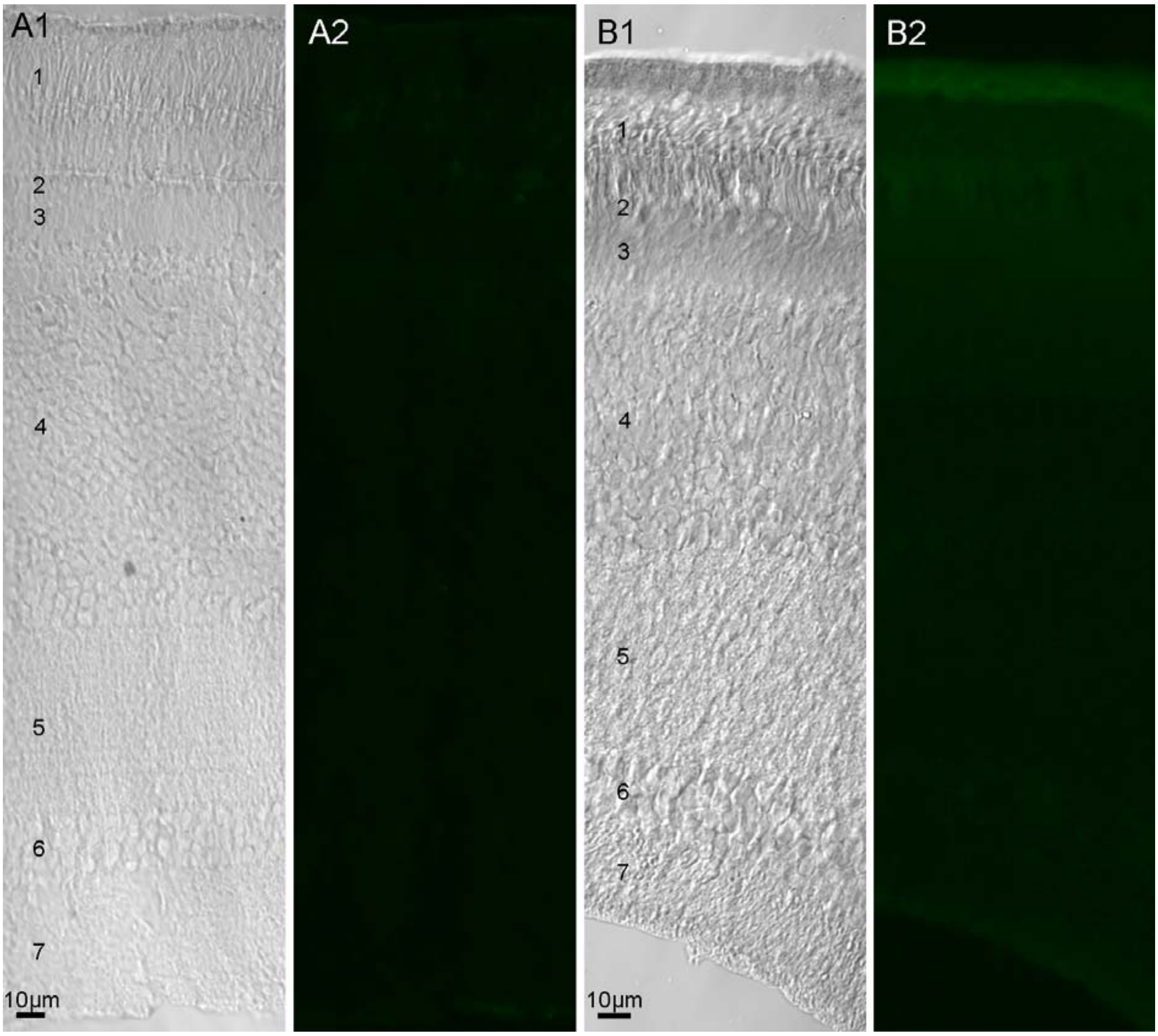

Fig. S2a. Pre-immune serum and peptide control for anti-Cry1a in a robin retina. (**A1**, **B1)** phase contrast image of the section indicating the different layers: 1 outer and inner segments of the photoreceptors with the oil droplets in between; 2 outer nuclear layer; 3 outer plexiform layer; 4 inner nuclear layer; 5 inner plexiform layer; 6 ganglion cell layer; 7 optic nerve fibre layer. (**A2**) control with pre-immune serum taken before immunising the animals, indicating that there was no unspecific reaction with other antibodies already present in the animals. (**B2**) control with the antibody and the specific peptide used to produce the antibody. The primary antiserum was blocked by mixing it with this peptide before applying it to the retina. Here, any remaining label would indicate that there are other antibodies in the serum that also bind to retinal structures.

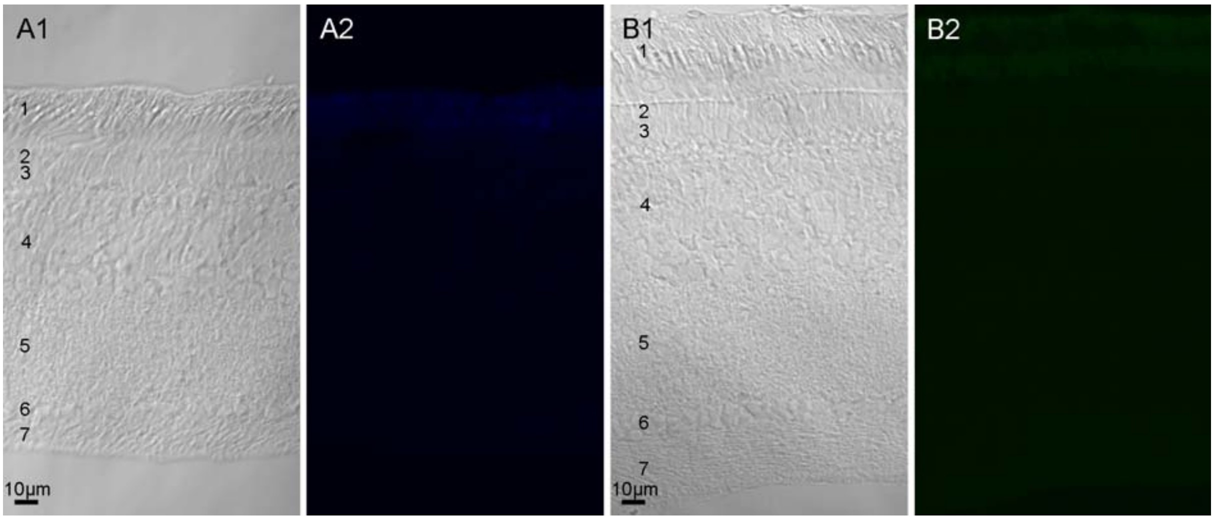

Fig. S2b. Control of specificity of secondary antibodies in a robin retina. Tissue incubation with only the secondary antibodies (omission of the primary antibodies from the protocol) showing that they reacted selectively with the primary antibodies. (**A1**, **B1**) phase contrast images indicating the retinal layers, see Figure S1; (**A2)** anti goat (cy3) and (**B2**) for anti guinea pig (cy5).

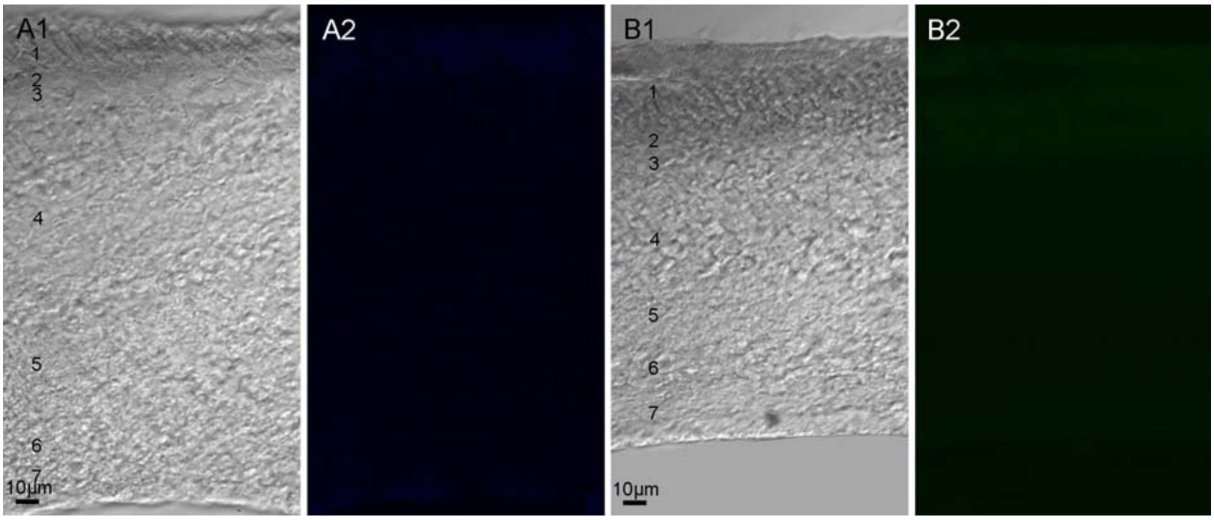

Fig. S2c. Control for cross-reactivity of the primary antibodies with inappropriate secondary antibodies in a robin retina. (**A1, B1**) phase contrast image indicating the retinal layers, see Figure S1. (**A2**) combining the primary antibody guinea pig anti-Cry1a with an anti-goat secondary antiserum; (**B2**) combining the primary antibody goat antiserum sc-14363 with an anti guinea pig secondary antiserum.
